# Frequency and factors associated with neuropathic pain in patients with knee osteoarthritis

**DOI:** 10.1016/j.ocarto.2026.100836

**Published:** 2026-06-11

**Authors:** Fulgence Kaboré, Abdoul Aziz, Ismael Ayouba Tinni, Charles Sougué, Camille Sompougdou, Aboubakar Ouédraogo, Enselme Zongo, Joelle S.W. Tiendrebeogo/Zabsonre, Dieu-Donné Ouédraogo

**Affiliations:** a Rheumatology Department. Bogodogo University Hospital. Ouagadougou. Burkina Faso; b Department of Internal Medicine. Souro Sanou University Hospital. Bobo-Dioulasso. Burkina Faso; c University Ledea-Bernard-OUEDRAOGO Ouahigouya. Burkina Faso

**Keywords:** Neuropathic pain, Risk factors, knee osteoarthritis, Sub-Saharan Africa

## Abstract

**Objective:**

To determine the frequency and factors associated with neuropathic pain in patients with knee osteoarthritis seen during rheumatology consultations.

**Methods:**

This was a descriptive and analytical cross-sectional study of patients with knee osteoarthritis who met the ACR criteria and who were seen in rheumatology consultations at a referral university hospital. A DN4 score greater than or equal to 4 was used to classify the pain as like neuropathic. Multivariable logistic regression analysis was used to determine adjusted ORs and 95% CI.

**Results:**

Three hundred and twenty-five patients with knee osteoarthritis were included in the study. The mean age of the patients was 57.85 ± 11.89 years. Neuropathic-like pain (DN4 ≥4) was identified in 32% of participants. Among DN4-positive individuals, the mean DN4 score was 6.51 ± 1.60 (range 4–10). The mean global WOMAC score was 30.98 ± 10.98. The mean score for pain under load according to the visual analog scale was 3.35 ± 1.376. After multivariable analysis, the associated factors with neuropathic-like pain were a history of knee trauma (OR = 4.525; 95%CI [1.497–13.678]; p = 0.007). Psychological history (OR = 9.941; 95%CI [3.066–32.235]; p < 0.001). Kellgren–Lawrence grades 3 and 4 (OR = 7.599; 95%CI [2.372–24.432]; p = 0.001) and WOMAC global score (OR = 1.093; 95%CI [1.053–1.134]; p < 0.001).

**Conclusion:**

A significant proportion of patients suffering from osteoarthritis of the knee experienced neuropathic-like pain, requiring the implementation of prevention and treatment strategies.

## 1 Introduction

Knee osteoarthritis is a frequent and disabling condition, posing management challenges due to the presence of multiple clinical manifestations as well as complex pathophysiological mechanisms [1,2]. Indeed, it is a multifactorial disease involving factors, such as aging, trauma, biomechanical loads, inflammation, and metabolic disturbances [3,4]. Worldwide estimates are that 9.6% of men and 18.0% of women aged ≥60 years have symptomatic knee osteoarthritis [3,4].

Pain is the most frequent symptom of knee osteoarthritis. The mechanisms of this pain are heterogeneous, which explains the variability in response to different treatments [5,6]. Determining the pain phenotype in patients with knee osteoarthritis helps to guide treatment strategies [5,6]. In osteoarthritis of the knee, pain may be nociceptive or neuropathic and may be inflammatory during congestive flare-ups [7]. It's important to identify neuropathic-like pain in knee osteoarthritis, because neuropathic-like pain is poorly controlled by conventional analgesics, such as non-steroidal anti-inflammatory drugs (NSAIDs). Patients with neuropathic-like pain are more likely to respond to non-standard analgesics such as tricyclic antidepressants, 5-hydroxytryptamine-norepinephrine reuptake inhibitors, or gabapentinoids than to NSAIDs [7,8].

Nociceptive pain is a mechanical pain caused by the degradation of cartilage and bone structure [6]. In contrast, neuropathic-like pain in knee osteoarthritis may be secondary to central and peripheral sensitization phenomena [6]. The peripheral sensitization phenomenon can be explained by the degradation of cartilage and bone structure, resulting in damage to intra-articular or periarticular nerve endings. For the central sensitization phenomenon, the mechanism begins with central sensitization and facilitation of neurons in the dorsal horn of the spinal cord [6,7]. Next, neuroplasticity occurs with the reorganization of somatosensory maps of the knee, leading to altered nociceptive message processing in the thalamus and sensorimotor cortex [6,7].

A number of screening tools have been developed to differentiate between neuropathic and non-neuropathic pain, including neuropathic-like pain in 4 (DN4) and pain DETECT. Although the DN4 identifies neuropathic pain features, it does not confirm lesion of the somatosensory nervous system. Increasingly, the term “nociplastic pain” is used in the osteoarthritis literature to describe pain arising from altered nociceptive processing without clear evidence of neural damage. In this study, we use the term “neuropathic-like pain” to reflect DN4-based classification [9].

The reported prevalence of neuropathic-like pain in patients with knee osteoarthritis ranges from 4% à 52%, depending on the study, and is associated with a significant deterioration in quality of life [[10], [11], [12]]. The highest prevalences have been reported in Spain (51.9%) and Turkey (44%); studies conducted in Japan have reported a low prevalence of approximately 4%–5.9% [12]. Few studies have been carried out in sub-Saharan Africa on this subject; in Cameroon, Central Africa, the frequency of neuropathic-like pain was 27.1% [13]. According to the literature, neuropathic-like pain was reported to be associated with age, female sex, body mass index (BMI), education level, occupational status, radiological severity of osteoarthritis, duration of symptoms, history of knee surgery, and presence of comorbid diseases [[13], [14], [15], [16]].

The diversity of knee osteoarthritis phenotypes according to African region could influence the prevalence of neuropathy and associated factors. We hypothesised that neuropathic-like pain (DN4 ≥4) in knee osteoarthritis would be associated with markers of structural severity (Kellgren-Lawrence stage), metabolic comorbidities (diabetes, BMI), prior joint trauma, and psychological distress, independent of age and sex. Therefore, the aim of our study was to determine the frequency and factors associated with neuropathic-like pain in patients with knee osteoarthritis seen in rheumatology consultation in a West African country to improve management.

## 2 Patients and methods

### 2.1 Design and period of study

This was a descriptive and analytical cross-sectional study of the frequency and factors associated with neuropathic-like pain during knee osteoarthritis in patients seen in rheumatology consultations at a university hospital in [Anonymized] from January 2023 to October 2023.

### 2.2 Study population

Patients were recruited during rheumatology consultations. The consultations were conducted by senior and junior rheumatologists. Each consultation was attended by approximately 20 patients.

Each patient underwent a complete clinical examination and a radiograph of the knees in frontal (weight-bearing), Schuss (weight-bearing), and lateral views. Axial incidence was not performed in all patients. Ultrasound and magnetic resonance imaging were not systematically performed. Blood counts, erythrocyte sedimentation rate, C-reactive protein (CRP) levels, and uric acid levels were measured in all patients. The search for germs, sodium urate crystals, and calcium pyrophosphate was performed in all patients with intra-articular effusion. All patients with diabetes mellitus were followed by an endocrinologist and had normal blood glucose levels and normal glycated hemoglobin for the previous 6 months. The diabetes specialist was responsible for screening his patients for diabetic neuropathy.

### 2.3 Inclusion criteria

Patients over 18 years of age with knee osteoarthritis who met the ACR criteria [17] and who agreed to participate in the study were included in the study. Knee osteoarthritis was diagnosed by a senior rheumatologist [K.F., T.JSW, O.DD].

### 2.4 Exclusion criteria

The study did not include any patients with radicular low back pain or those with neurological pathologies likely to cause neuropathic-like pain such as trauma, brain and spinal cord injuries. Patients with clinically diagnosed diabetic neuropathy, dementia or severe cognitive impairment, those who had difficulty assessing pain, those who were unable to complete questionnaires, and those who refused to participate were not included in the study.

### 2.5 Sample size

The sample size was calculated according to the Schwartz formula, with a frequency of 27.1% from Lekpa et al. (Cameroon, 2020) study [13], a 1.96 for Z value, and a precision of 5% [13], so the minimum sample size was 304. During the study period, 325 patients met the inclusion criteria and were included in the study. Our sample was not adjusted for non-responders.

### 2.6 Study variables

Neuropathic-like pain was assessed using the DN4 (neuropathic-like pain in 4) questionnaire (Supplementary Material 1), which comprises 10 items: 7 questions on the sensory characteristics of pain and 3 items from the clinical examination, to detect the presence or absence of allodynia and hypoesthesia to touch [6]. Each item that is answered with "yes" is worth one point; a score of 4 or more is indicative of neuropathic pain. The DN4 has demonstrated sensitivity of approximately 82–90% and specificity of 80–94% for neuropathic pain. It has been used in knee osteoarthritis populations in prior studies [6]. The Western Ontario and McMaster Universities Arthritis Index (WOMAC) has been employed to determine the functional impact of knee osteoarthritis [18]. It focuses on three themes: pain, stiffness and knee function. The pain subscale is assessed on a total of 20 points (≤10 points: mild to moderate pain; >10 points: severe to maximal pain). Joint stiffness is assessed on an 8-point scale (≤4 points: mild to moderate stiffness; >4 points: severe to maximal stiffness) [18]. Pain at rest, under load, or in motion was assessed using a visual analog scale, with scores ranging from 0 to 10. Other variables included patient sociodemographics, psychological history (depression, anxiety, stress) and comorbidities was self-reported physician-diagnosed, and clinical and radiological features of knee osteoarthritis. The severity of knee osteoarthritis was assessed using the Kellgren-Lawrence scale based on standard X-rays [19].

### 2.7 Data analysis

All statistical analyses were performed using R software version 4.2.3 (R Foundation for Statistical Computing, Vienna, Austria). A two-sided p-value <0.05 was considered statistically significant.

Continuous variables were examined for normality using graphical methods and the Shapiro–Wilk test. Normally distributed variables were summarized as mean ± standard deviation, whereas non-normally distributed variables were presented as median and interquartile range. Categorical variables were described as frequencies and percentages.

The primary outcome was neuropathic-like pain defined by a DN4 score ≥4 (binary variable). Participant characteristics were first described for the overall sample and then compared according to DN4 status (DN4 <4 vs ≥ 4). Between-group comparisons were performed using Student's t-test or Mann–Whitney *U* test for continuous variables, as appropriate, and chi-square test or Fisher's exact test for categorical variables.

Univariable logistic regression analyses were conducted to estimate the crude odds ratios and 95% confidence intervals for the association between each independent variable and neuropathic-like pain.

A multivariable logistic regression model was then constructed a priori based on clinical relevance and previous literature. The following variables were included irrespective of their univariable significance: age, sex, BMI, type 2 diabetes mellitus, history of knee trauma, psychological history (self-reported physician-diagnosed depression, anxiety, or stress disorder), radiographic severity (Kellgren–Lawrence grade), and global WOMAC score. These variables were selected to account for potential confounding and to test the study hypothesis regarding structural severity, metabolic comorbidities, prior joint injury, and psychological factors.

Continuous variables (age, BMI, WOMAC score) were entered into the model as continuous predictors to preserve statistical power and avoid information loss. Kellgren–Lawrence grade was modeled as an ordinal variable.

Multicollinearity was assessed using variance inflation factors, with values > 5 considered indicative of significant collinearity. Model fit was evaluated using the Hosmer–Lemeshow goodness-of-fit test. This gave us the adjusted odds ratios.

### 2.8 Ethical considerations

The protocol was approved by the institutional ethics committee, by deliberation N° 2023-01-007. Administrative authorization for data collection was also obtained from the management of the university hospital. Confidentiality and anonymity (no identifiable data were stored in the analytical dataset) were respected during data collection and processing, in accordance with the Helsinki recommendations. Written consent was obtained from all participants before inclusion.

## 3 Results

### 3.1 Sociodemographic, clinical and radiological characteristics

A total of 325 patients with symptomatic knee osteoarthritis were included in the study. The mean age of the patients was 57.85 ± 11.89 years, with extremes of 25 and 88 years. Two hundred and sixty-seven (82.2%) patients were female, resulting in a sex ratio of 0.21. The mean BMI of the patients was 29.56 kg/m^2^ ± 6.02 kg/m^2^, with extremes of 18.22 kg/m^2^ and 55.77 kg/m^2^. One hundred nine patients (33.5%) had arterial hypertension, and 29 patients (8.9%) had diabetes mellitus type 2.

The mean duration of disease progression was 4.25 years ±3.72 years, with a minimum of one year and a maximum of 13 years. Radiographically, 241 (74.2%) patients had advanced structural disease (Kellgren Lawrence grade ≥3), and 212 (65.2%) had bilateral involvement. Table 1 presents the distribution of patients included in the study according to sociodemographic, clinical, and radiological characteristics, based on DN4 status.

**Table 1:** Participant characteristics overall and by DN4 status

Variables	DN4 status	
	Negative (<4) mean ± SD	Positive (≥4) mean ± SD
**Age**	57.20 ± 12.11	59.19 ± 11.35
**BMI**	28.77 ± 5.06	31.20 ± 7.39
**WOMAC pain**	5.76 ± 2.16	8.24 ± 2.12
**WOMAC function**	18.98 ± 6.96	27.38 ± 7.07
**WOMAC stiffness**	2.47 ± 0.90	3.26 ± 0.99
**WOMAC global**	27.21 ± 9.49	38.88 ± 9.64
**VAS at rest**	2.0 ± 0.91	2.58 ± 1.34
**VAS on load**	3.06 ± 1.20	3.94 ± 1.52

	Negative (<4)	Positive (≥4)

N (%)	N (%)
**Gender**		
Female	183 (68.5)	52 (29.9)
Male	37 (63.8)	53 (35.1)
**Hypertension**		
No	149 (69.0)	67 (31.0)
Yes	71 (65.1)	38 (34.9)
**Diabetes mellitus**		
No	205 (69.3)	91(30.7)
Yes	15 (51.7)	14 (48.3)
**Gastro-Duodenal Ulcer**		
No	168 (71.2)	68 (28.8)
Yes	52 (58.4)	37 (41.6)
**Knee Surgery**		
No	165 (70.5)	69 (29.5)
Yes	55 (60.4)	36 (39.6)
**Knee traumatism**		
No	208 (71.0)	85 (29.0)
Yes	12 (37.5)	20 (62.5)
**Psychological history**		
No	214 (72.3)	82 (27.7)
Yes	6 (20.7)	23 (79.3)
**Knee puncture**		
No	149 (74.5)	51 (25.5)
Yes	71 (56.8)	54 (43.2)
**Location of osteoarthritis of the knee**		
Bilateral	140 (66.0)	72 (34.0)
Right	51 (73.9)	18 (26.1)
Left	29 (65.9)	15 (34.1)
**Type of knee osteoarthritis**		
Tricompartmental	129 (66.2)	66 (33.8)
Tibiofemoral	79 (67.5)	38 (32.5)
Patellofemoral	12 (92.3)	1 (7.7)
**Kellgren-Lawrence grade**		
Grades 1 and 2	74 (92.5)	6 (7.5)
Grades 3 and 4	146 (59.6)	99 (40.4)
**DN4 components**		
Burns (Yes)	1 (1.6)	62 (98.4)
Electric shock (Yes)	8 (8.7)	84 (91.3)
Painful cold sensation (Yes)	5 (6.6)	71 (93.4)
Tingling (Yes)	18 (15.9)	95 (84.1)
Prickling sensation (Yes)	9 (8,1)	102 (91.9)
Numbness (Yes)	7 (7.2)	90 (92.8)
Itching (Yes)	6 (8.1)	68 (91.9)
Hypoesthesia to touch (Yes)	1 (11.1)	8 (88.9)
Hypoesthesia to stinging (Yes)	2 (16.7)	10 (83.3)
Pain on rubbing (Yes)	169 (64.3)	94 (35.7)

WOMAC: Western Ontario and McMaster Universities Osteoarthritis Index VAS: Visual Analog Scale

∗: Human immunodeficiency virus infection, sickle cell anemia, hepatitis B virus infection, asthma, kidney failure. BMI: Body Mass Index DN4: Neuropathic like-Pain in 4

Characteristics of patients with neuropathic pain, WOMAC score and visual analog scale score:

Neuropathic-like pain (DN4 ≥4) was identified in 32% of participants. Among DN4-positive individuals, the mean DN4 score was 6.51 ± 1.60 (range 4–10).

The mean global WOMAC score was 30.98 ± 10.98, with extremes of 3 and 60. The mean WOMAC pain score was 6.56 ± 2.44, with extremes of 1 and 13. The mean WOMAC stiffness score was 2.72 ± 1.00, with extremes of 1 and 6. The mean WOMAC function score was 21.50 ± 8.02, with extremes of 1 and 42.

The mean resting visual analog scale pain score was 2.19 ± 1.10, with extremes of 1 and 8. The mean pain score according to the visual analog scale under load was 3.35 ± 1.37, with extremes of 1 and 10 (Table 1).

### 3.2 Factors associated with neuropathic pain

In univariable logistic regression analyses, neuropathic-like pain was significantly associated with BMI, knee trauma, psychological history, Kellgren-Lawrence grade, WOMAC global, VAS at rest and VAS on load (Table 2). Disease duration and diabetes were not significantly associated with DN4 status.

**Table 2:** Unadjusted logistic regression

Variables	DN4(Positive)		
	Unadjusted OR	95% CI	P Value
**Age**	1.014	[0.99–1.03]	0.16
**BMI**	1.068	[1.027–1.11]	0.001
**Gender**			
Female	Ref	–	0.484
Male	1.236	[0.68–2.24]	
**Diabetes mellitus type 2**			
No	Ref	–	0.058
Yes	2.103	[0.974–4.537]	
**Knee traumatism**			
No	Ref	–	<0.001
Yes	4.078	[1.910–8.711]	
**Psychological history**			
No	Ref	–	<0.001
Yes	10.004	[3.932–25.453]	
**Kellgren-Lawrence grade**			
Grades 1 and 2	Ref	–	<0.001
Grades 3 and 4	8.363	[3.503–19.966]	
**WOMAC Global**	1.128	[1.094–1.162]	<0.001
**VAS at rest**	1.597	[1.272–2.006]	<0.001
**VAS on load**	1.612	[1.335–1.946]	<0.001

OR: odds ratio CI: confidence interval

In the multivariable logistic regression model adjusted for age, sex, BMI, diabetes, knee trauma, psychological history, KL grade, and WOMAC global score:

History of knee trauma was independently associated with neuropathic-like pain (OR = 4.525; 95%CI [1.497–13.678]; p = 0.007). Psychological history was independently associated (OR = 9.941; 95%CI [3.066–32.235]; p < 0.001). Kellgren–Lawrence grades 3 and 4 was associated with neuropathic-like pain (OR = 7.599; 95%CI [2.372–24.432]; p = 0.001). Each one-grade increase in WOMAC global score was was independently associated (OR = 1.093; 95%CI [1.053–1.134]; p < 0.001). No significant association was observed with disease duration or diabetes. Table 3 shows the risk factors associated with neuropathic-like pain in patients with knee osteoarthritis during the study period at the university hospital. The variance inflation factors were less than 5. The Hosmer-Lemeshow test showed that the model was well adjusted.

**Table 3:** adjusted logistic regression multivariable

Variables	DN4(Positive)		
	Adjusted OR	95% CI	P Value
**Age**	0.983	[0.954–1.013]	0.267
**BMI**	1.017	[0.962–1.074]	0.556
**Gender**			
Female	Ref	–	0.180
Male	1.775	[0.768–4.107]	
**Diabetes mellitus type 2**			
No	Ref	–	0.116
Yes	2.218	[0.821–5.983]	
**Knee traumatism**			
No	Ref	–	0.007
Yes	4.525	[1.497–13.678]	
**Psychological history**			
No	Ref	–	<0.001
Yes	9.941	[3.066–32.235]	
**Kellgren-Lawrence grade**			
Grades 1 and 2	Ref	–	0.001
Grades 3 and 4	7.599	[2.372–24.432]	
**WOMAC Global**	1.093	[1.053–1.134]	<0.001
**VAS at rest**	0.840	[0.494–1.428]	0.520
**VAS on load**	1.564	[0.996–2.456]	0.052

OR: odds ratio CI: confidence interval

## 4 Discussion

In this cross-sectional study of patients with symptomatic knee osteoarthritis, neuropathic-like pain was present in nearly one-third of participants. Independent associations were identified with prior knee trauma, psychological history, greater radiographic severity and WOMAC global score, while disease duration and diabetes were not significantly associated.

This frequency of neuropathic-like pain is in line with that reported in the literature [10,12].

The mechanisms underlying neuropathic-like pain remain poorly understood. However, peripheral nerve damage due to injury or local inflammation has been implicated in the development of this condition [20]. Conversely, it is possible that neuropathic-like pain occurs in association with damage to nerves innervating the subchondral bone due to its load-bearing surface in advanced osteoarthritis [11]. The characterization of pain in osteoarthritis of the knee is therefore important for the appropriate management of the patient [21,22]. It is important to look for neuropathic-like pain in patients who present with pain that is initially relieved by analgesics and NSAIDs, but becomes more persistent, widespread, and refractory as the disease progresses. At this stage, centrally acting agents such as duloxetine and pregabalin may be effective [[21], [22], [23], [24]].

A history of knee trauma emerged as a strong independent correlate of neuropathic-like pain. Joint injury may lead to peripheral nerve damage, altered joint biomechanics, and sustained nociceptive activation. These mechanisms could predispose patients to maladaptive neuroplastic changes and the development of neuropathic-like features. This finding highlights the importance of recognizing post-traumatic OA as a potentially distinct pain phenotype [14].

Psychological disorders are associated with neuropathic-like pain in patients with knee osteoarthritis. This association has been found in previous studies [[14], [15], [16],[25], [26], [27], [28], [29]]. Importantly, although some DN4 descriptors may overlap with symptoms observed in mood disorders, the persistence of this association after multivariable adjustment suggests a genuine interaction between psychological factors and altered pain processing in knee OA [30,31]. Furthermore, it is possible that pain in patients with knee osteoarthritis contributes to functional limitations, fatigue, and possible sleep disturbances, thus contributing to decreased mood, increased anxiety and worsening pain and functional scores, resulting in a vicious cycle [2,7,20,32].

The independent association between higher Kellgren–Lawrence grade and neuropathic-like pain supports the hypothesis that advanced structural damage may contribute to altered nociceptive processing. Subchondral bone remodeling, bone marrow lesions, and synovial inflammation have been shown to increase peripheral nociceptive input, potentially facilitating central sensitization mechanisms. Our findings suggest that structural progression in knee OA may not only reflect mechanical deterioration but also a shift toward more complex pain phenotypes [10,21].

In contrast to our results, several studies have not found an association between neuropathic-like pain and the radiologic stage of the knee osteoarthritis [[33], [34], [35]]. The relationship between the severity of osteoarthritis and pain on radiographic images remains undetermined. Many patients have radiographic signs of osteoarthritis in the absence of pain, while others have few radiographic signs of osteoarthritis but moderate to severe pain [36]. However, some authors have found an association between neuropathic-like pain and higher clinical pain associated with a lower Kellgren-Lawrence score. This association may be explained by the phenomenon of central sensitization [37]. These authors suggest that central sensitization may be an endophenotypic marker of chronic pain in knee OA patients who report high levels of clinical pain in the absence of moderate to severe radiographic evidence of pathology. This may help to explain the oft-reported phenomenon of a minimal to moderate relationship between self-reported pain and radiographic evidence of pathology in osteoarthritis [37].

In our series, we did not observe any link between type 2 diabetes mellitus and neuropathic-like pain [14]. The presence of preexisting diabetic neuropathy increases the likelihood of developing neuropathic-like pain in patients with knee osteoarthritis [38]. Interestingly, disease duration was not associated with neuropathic-like pain. This finding suggests that neuropathic-like features may emerge early in the disease course rather than representing a late-stage complication. It reinforces the concept that pain phenotype in OA is not solely time-dependent but influenced by structural, traumatic, and psychosocial factors [14].

## 5 Clinical implications

Identifying neuropathic-like pain features in knee OA has important therapeutic implications. Patients with DN4 positivity may respond differently to conventional analgesics targeting nociceptive mechanisms. Recognition of this phenotype could support more individualized treatment strategies, including centrally acting agents or multidisciplinary pain management approaches [[22], [23], [24],^38^].

## 6 Strengths and limitations

Strengths of this study include the use of a validated neuropathic pain questionnaire, systematic radiographic assessment, and multivariable modeling based on an a priori hypothesis.

However, several limitations should be acknowledged. The cross-sectional design precludes causal inference. Quantitative sensory testing was not performed, limiting mechanistic interpretation. Psychological factors were based on self-report and may be subject to misclassification. Finally, the single-center design may limit generalisability.

## 7 Conclusion

Neuropathic-like pain was present in nearly one-third of patients with symptomatic knee osteoarthritis and was independently associated with prior knee trauma, psychological history, and greater radiographic severity. These findings support the concept that knee osteoarthritis pain is heterogeneous and not solely driven by nociceptive mechanisms related to structural damage. Importantly, neuropathic-like features were not associated with disease duration, indicating that altered pain phenotypes may emerge early in the disease course rather than representing a late complication. Prospective longitudinal studies integrating quantitative sensory testing and imaging biomarkers are warranted to further elucidate the temporal and mechanistic pathways underlying neuropathic-like pain in knee osteoarthritis.

## Author contributions

F.K, A-A, I.A.T, C.S, and C.S participated in the design of the study and coordinated the participation of clinical investigators. A.O, E.Z, J.S.W T/Z and D-D.O participated in the design of the study and performed the analysis of data. All authors participated in the review and extraction of literature, interpretation of results, and preparation of the manuscript. All authors had full access to the data and have provided their final approval of the version of the manuscript to be submitted for publication.

## Data availability statement

Data are available from the authors upon reasonable request.

## Declaration of Generative AI in scientific writing

None.

## Role of the funding source

The authors did not receive any funding to conduct this study.

## Conflict of interest

None of the authors have a conflict of interest to disclose.
