# Supplementary material for: Frequency and factors associated with neuropathic pain in patients with knee osteoarthritis
Source: Osteoarthr Cartil Open. 2026 Jun 11;8(3):100836. doi: 10.1016/j.ocarto.2026.100836 (PMC13314881; doi:10.1016/j.ocarto.2026.100836)
Supplement: Multimedia component 1 [file mmc1.docx]

| Variables | Number | Percentage |
| --- | --- | --- |
| **Age (years)**  ≤60  >60 | 174  151 | 53.5  46.5 |
| **Gender**  Female  Male | 267  58 | 82.2  17.8 |
| **Marital status**  Married  Not married | 245  80 | 75.4  24.6 |
| **Education level**  Uneducated  Educated | 178  147 | 54.8  45.2 |
| **Profession**  Not employed  Employee | 273  52 | 84.0  16.0 |
| **Residence**  Urban  Rural | 226  99 | 69.5  30.5 |
| **Hypertension**  **Diabetes mellitus**  **Gastro-Duodenal ulcer**  **Knee surgery**  **Knee traumatism**  **Psychological**  **No knee swelling**  **Family arthritis**  **Knee puncture**  **Other*** | 109  29  89  91  32  29  56  92  125  24 | 33.5  8.9  27.4  28.0  9.8  8.9  17.2  28.3  38.5  7.4 |
| **BMI (kg/m2)**  Normal  Abnormal | 70  255 | 21.5  78.5 |
| **Location of osteoarthritis of the knee**  Bilateral  Right  Left | 212  69  44 | 65.2  21.2  13.5 |
| **Type of knee osteoarthritis**  Tricompartmental  Tibiofemoral  Patellofemoral | 195  117  13 | 60  36  4 |
| **Kellgren-Lawrence Stadium**  Stage 1  Stage 2  Stage 3  Stage 4 | 2  78  241  4 | 0.6  24.0  74.2  1.2 |
| **DN4**  < 4  ≥ 4 | 220  105 | 67.7  32.3 |
| **DN4 components**  Burns  Electric shock  Painful cold sensation  Tingling  Prickling sensation  Numbness  Itching  Hypoesthesia to touch  Hypoesthesia to stinging  Pain on rubbing | 63  92  76  113  111  97  74  9  12  263 | 19.4  28.3  23.4  34.8  34.2  29.8  22.8  2.8  3.7  80.9 |
| **WOMAC pain**  ≤10  >10  **WOMAC Function**  ≤24  >24  **WOMAC Stiffness**  ≤4  >4  **WOMAC Global**  ≤50  >50 | 304  21  204  121  314  11  307  18 | 93.5  6.5  62.8  37.2  96.6  3.4  94.5  5.5 |
| **VAS at rest**  ≤5  >5 | 320  5 | 98.5  1.5 |
| **VAS on load**  ≤5  >5 | 299  26 | 92.0  8.0 |
| **DN4**  < 4  ≥ 4 | 220  105 | 67.7  32.3 |

*: Human immunodeficiency virus infection, sickle cell anemia, hepatitis B virus infection, asthma, kidney failure.

BMI: Body Mass Index

DN4: Neuropathic Pain in 4

WOMAC: Western Ontario and McMaster Universities Osteoarthritis Index

VAS: Visual Analog Scale
