# Supplementary material for: Frequency and factors associated with neuropathic pain in patients with knee osteoarthritis
Source: Osteoarthr Cartil Open. 2026 Jun 11;8(3):100836. doi: 10.1016/j.ocarto.2026.100836 (PMC13314881; doi:10.1016/j.ocarto.2026.100836)
Supplement: Multimedia component 2 [file mmc2.docx]

**Data Collection Form**

**IDENTIFICATION AND SOCIODEMOGRAPHIC DATA**

**1.1 Date of First Consultation**

**/**/____

**1.2 Sex**

Male = 1
Female = 2

**1.3 Date of Birth**

**/**/____
or
Age: ______ years

**1.4 Where do you live?**

Urban area
Rural area

**1.5 Participant’s Occupation**

Retired = 1
Housewife = 2
Farmer = 3
Trader = 4
Unemployed = 5
Other = 6 (Specify): _______________________

**1.6 If retired, occupation before retirement:**

**1.7 Educational Level**

Never attended school = 1
Primary = 2
Secondary (6th–9th grade) = 3
Secondary (10th–12th grade) = 4
University = 5

**1.8 Marital Status**

Married = 1
Separated or divorced = 2
Widowed = 3
Cohabiting = 4
Single = 5
Other = 6 (Specify): _______________________

**HISTORY OF THE DISEASE**

**2.1 Characteristics of Knee Osteoarthritis**

Specify type:
Bilateral: ___
Left: ___
Right: ___

Age at onset: ______

Mode of onset:
Sudden ___
Progressive ___

Pain intensity (VAS):
At rest: ___ /10
On movement: ___ /10

Duration of pain progression: ____ days

**2.2 General Symptoms**

Yes ___ No ___

If yes, specify:
Fever ___
Anorexia ___
Asthenia ___
Weight loss ___

**2.3 Visceral Symptoms**

Yes ___ No ___

If yes, specify: ____________________

**MEDICAL HISTORY AND COMORBIDITIES**

**3.1 Hypertension (treated or untreated)?**

Yes ___
No ___
I don’t know ___

If yes:
Duration: ___ years
Current treatment: __________________

**3.2 Myocardial Infarction?**

Yes ___
No ___
I don’t know ___

**3.3 Stroke?**

Yes ___
No ___
I don’t know ___

**3.4 Diabetes Mellitus?**

Yes ___
No ___
I don’t know ___

If yes, treatment taken:
Insulin ___
Oral antidiabetic drugs ___ (Specify: ____________)

**3.5 Renal Failure?**

Yes ___
No ___
I don’t know ___

If yes:
Duration: ___ years
Are you on hemodialysis? Yes ___ No ___

**3.6 Hypercholesterolemia (treated or untreated)?**

Yes ___
No ___
I don’t know ___

**3.7 Asthma?**

Yes ___
No ___
I don’t know ___

**3.8 Sickle Cell Disease?**

Yes ___
No ___
I don’t know ___

**3.9 Gastro-duodenal Ulcer?**

Yes ___
No ___
I don’t know ___

**3.10 Menopause**

Yes ___
No ___

If yes, year of menopause: __________

**3.11 History of Surgery (including knee prosthetic surgery)**

Yes ___
No ___
I don’t know ___

If yes, specify type and year: ___________________

**TRIGGERING FACTORS AND LIFESTYLE**

**4.1 Occupational, sports-related or road traffic trauma**

(knee fracture, sprain, dislocation, meniscal injury)

Yes ___
No ___

**4.2 Repeated minor trauma**

Yes ___
No ___

**4.3 Other Osteoarthritis Location**

Yes ___
No ___

If yes, specify site(s): __________________

**4.4 Family History of Osteoarthritis**

Yes ___
No ___

**4.5 Cardiovascular Risk Factors**

Yes ___
No ___

If yes, specify:

Excess alcohol consumption?
Yes ___ No ___
If yes, duration: ___ years

Smoking?
Yes ___ No ___
If yes: duration ___ years
Pack-years: ___

Lipid profile (total cholesterol, LDL, HDL, triglycerides)?
Yes ___ No ___

**5 – PHYSICAL EXAMINATION**

**5.1 Parameters**

Weight: ____ kg
Height: ____ cm
BMI: ____ kg/m²

**5.2 Lower Limb Architectural Abnormalities**

Axial deviations:
Yes ___ No ___

If yes, specify type:

Frontal deviations:
Genu varum ___
Genu valgum ___

Sagittal deviation:
Genu flexum ___
Genu recurvatum ___
Other (specify): ___________

**5.3 Foot Abnormalities**

Yes ___ No ___

If yes, specify type:
Clubfoot ___
Valgus foot ___
Talus foot ___
Other ___ (Specify: ________)

**5.4 Quadriceps circumference**

Yes ___ No ___

Right knee: ___
Left knee: ___

**5.5 WOMAC Score (Western Ontario and McMaster Universities Index)**

**5.6 DN4 Questionnaire**

**COMPLEMENTARY EXAMINATIONS**

**6.1 Laboratory Tests**

**6.1.1 Synovial Fluid**

Mechanical ___
Inflammatory ___
Not available ___

**6.1.2 Other biological abnormalities**

Yes ___
No ___

If yes, specify: ____________________

**6.2 Imaging**

**6.2.1 Kellgren and Lawrence Radiological Stage**

**6.2.2 Other radiological abnormalities**

Yes ___
No ___

If yes, specify: ____________________
