# Supplementary material for: Frequency and factors associated with neuropathic pain in patients with knee osteoarthritis
Source: Osteoarthr Cartil Open. 2026 Jun 11;8(3):100836. doi: 10.1016/j.ocarto.2026.100836 (PMC13314881; doi:10.1016/j.ocarto.2026.100836)
Supplement: Multimedia component 3 [file mmc3.pdf]

DN4 – QUESTIONNAIRE

To estimate the probability of neuropathic pain, please answer yes or no for each item of the following four questions.

INTERVIEW OF THE PATIENT

QUESTION 1:

Does the pain have one or more of the following characteristics?	YES	NO
Burning	❑	❑
Painful cold	❑	❑
Electric shocks	❑	❑

QUESTION 2:

Is the pain associated with one or more of the following symptoms in the same area?	YES	NO
Tingling	❑	❑
Pins and needles	❑	❑
Numbness	❑	❑
Itching	❑	❑

EXAMINATION OF THE PATIENT

QUESTION 3:

Is the pain located in an area where the physical examination may reveal one or more of the following characteristics?	YES	NO
Hypoesthesia to touch	❑	❑
Hypoesthesia to pinprick	❑	❑

QUESTION 4:

In the painful area, can the pain be caused or increased by:	YES	NO
Brushing?	❑	❑

YES = 1 point
NO = 0 points

Patient's Score: /10

https://www.en.wikimedecine.fr
